# Involvement of amygdala–prefrontal dysfunction in the influence of negative emotion on the resolution of cognitive conflict in patients with schizophrenia

**DOI:** 10.1002/brb3.1064

**Published:** 2018-07-13

**Authors:** Jaesub Park, Ji‐Won Chun, Hae‐Jeong Park, Eosu Kim, Jae‐Jin Kim

**Affiliations:** ^1^ Institute of Behavioral Science in Medicine Yonsei University College of Medicine Seoul Korea; ^2^ Department of Psychiatry National Health Insurance Service Ilsan Hospital Goyang Korea; ^3^ Department of Psychiatry Seoul St. Mary's Hospital The Catholic University of Korea College of Medicine Seoul Korea; ^4^ Department of Nuclear Medicine Yonsei University College of Medicine Seoul Korea; ^5^ Department of Psychiatry Yonsei University College of Medicine Seoul Korea

**Keywords:** amygdala, cognitive conflict, negative emotion, prefrontal cortex, schizophrenia

## Abstract

**Introduction:**

Patients with schizophrenia often have impaired cognition and abnormal conflict control. Conflict control is influenced by the emotional values of stimuli. This study investigated the neural basis of negative emotion interference with conflict control in schizophrenia.

**Methods:**

Seventeen patients with schizophrenia and 20 healthy controls underwent functional magnetic resonance imaging while performing the emotional Simon task, in which positive or negative emotional pictures were located in congruent or incongruent positions. Analysis was focused on identifying brain regions with the significant interaction among group, emotion, and conflict in whole brain voxel‐wise analysis, and abnormality in their functional connectivity in the patient group.

**Results:**

The regions showing the targeted interaction was the right amygdala, which exhibited significantly reduced activity in the negative congruent (*t *=* *−2.168, *p *=* *0.036) and negative incongruent (*t *=* *−3.273, *p *=* *0.002) conditions in patients versus controls. The right amygdala also showed significantly lower connectivity with the right dorsolateral prefrontal cortex in the cognitive and emotional loading contrast (negative incongruent—positive congruent) in patients versus controls (*t *=* *−5.154, *p *<* *0.01), but not in the cognitive‐only or emotional‐only loading contrast.

**Conclusions:**

These results suggest that negative emotion interferes with cognitive conflict resolution in patients with schizophrenia due to amygdala–dorsolateral prefrontal cortex disconnection. Based on these findings, interventions targeting conflict control under negative emotional influence may promote cognitive rehabilitation in patients with schizophrenia.

## 1 INTRODUCTION

Executive dysfunction causes functional impairments in patients with schizophrenia (Green, Kern, Braff, & Mintz, 2000) and is an important target for cognitive rehabilitation for the affected patients (Penadés et al., 2010). Executive function includes conflict control, the ability to select and execute a goal‐directed response to situations involving conflict produced by competing stimuli or responses (Botvinick, Braver, Barch, Carter, & Cohen, 2001). The ability to resolve cognitive conflict is crucial in our social environment in which we are often forced to choose between competing options.

The influence of emotion on conflict control has been in the research spotlight. When situations causing cognitive conflict are repeated, the speed of the cognitive processes is reduced, particularly in response to negative stimuli (Padmala, Bauer, & Pessoa, 2011). Patients with schizophrenia have difficulty with conflict control including a cognitive inhibition deficit in Stroop interference (Laurenson et al., 2015; Westerhausen, Kompus, & Hugdahl, 2011). Conflict control appears to be especially disturbed under negative emotional influence in schizophrenia.

The prefrontal cortex (PFC), including the dorsolateral prefrontal cortex (DLPFC) and anterior cingulate cortex (ACC), plays a central role in the top–down processes of conflict control (Chun, Park, Kim, Kim, & Kim, 2017; Miller, 2000; Stokes et al., 2013). Negative emotion reduces PFC activity to a greater extent than neutral or positive emotion (Sommer, Hajak, Döhnel, Meinhardt, & Müller, 2008). Amygdala–PFC interactions are involved in emotional conflict, and top–down inhibition of the amygdala by the ACC is important in resolving emotional conflict (Etkin, Egner, Peraza, Kandel, & Hirsch, 2006). Amygdala–PFC connectivity increases during cognitive conflict resolution was in subjects with high levels of trait or state anxiety (Bijsterbosch, Smith, & Bishop, 2015; Krug & Carter, 2010). Abnormal neural responses in the DLPFC, ACC, and amygdala during cognition–emotion interactions have also been observed in patients with schizophrenia (Anticevic, Repovs, & Barch, 2012; Park, Park, Chun, Kim, & Kim, 2008; Ursu et al., 2011).

Disruption of neural circuits connecting the amygdala and PFC may play a role in abnormal conflict control under negative emotion in schizophrenia. Amygdala–prefrontal connectivity is reduced in patients with schizophrenia during emotion perception (Bjorkquist, Olsen, Nelson, & Herbener, 2016) or emotional distractors (Mukherjee et al., 2016), and during bottom–up emotional processes (Comte et al., 2017). However, the tasks used in previous studies have involved emotional rather than cognitive conflict. A few studies have focused on emotion in conflict control in patients with schizophrenia. Patients with schizophrenia have exhibited difficulty resolving cognitive conflict under negative emotion and reduced DLPFC activity versus healthy controls (Tully, Lincoln, & Hooker, 2014), but prefrontal–amygdala connectivity has not been addressed.

In this study, a task provoking the Simon effect was used to address cognitive conflict in patients with schizophrenia. The Simon effect refers to the finding that reactions tend to be faster and more accurate when a stimulus is presented in the same relative location as a response (Simon, 1990). To explore the effect of emotion on cognitive conflict resolution, we employed a modified Simon task with emotional pictures (Chun et al., 2017).

We investigated brain abnormality in the relationship between emotion and conflict control in patients with schizophrenia using functional magnetic resonance imaging (fMRI). We aimed to identify brain regions with abnormal activity during task performance and to analyze the association between abnormal regional activities and clinical symptoms. In addition, we attempted to determine whether functionally abnormal regions show altered connectivity. We hypothesized that patients would show abnormal prefrontal–amygdala activity and connectivity in response to emotional interferences when performing cognitive conflict‐resolving tasks.

## 2 MATERIALS AND METHODS

### 2.1 Participants

The study included 17 patients with schizophrenia (7 male/10 female) and 20 healthy controls (9 male/11 female). Patients were recruited from a psychiatric outpatient clinic, and healthy controls were recruited using open advertisement on the internet. Exclusive diagnosis of schizophrenia in patients and assessment of mental health status in controls were based on the Structural Clinical Interview for DSM‐IV (First, Gibbon, Spitzer, & Williams, 1996). Additional exclusion criteria included (a) neurological or significant medical illness, (b) current or past substance abuse or dependence, and (c) left handedness. There was no significant statistical difference in gender, age, or years of education between the patient and control groups (Table 1). Intelligence was estimated using the Raven's Progressive Matrices (RPM) score (Raven, Court, & Raven, 1988) and was significantly lower in patients than in controls (*t *=* *2.725, *p *=* *0.01). Patients’ symptom severity was measured using the Positive and Negative Syndrome Scale (PANSS) (Kay, Fiszbein, & Opler, 1987). All patients were medicated with fixed‐dose antipsychotics for at least 3 months before the time of testing. All participants gave written informed consent, and all procedures were approved by the local Institutional Review Board.

**Table 1:** Demographic and clinical characteristics of participants and their behavioral performances

	Schizophrenia (*N* = 17)	Healthy controls (*N* = 20)	*t*	*p*
Demographic variables				
Sex (male/female)	7/10	9/11		
Age (years)	27.2 ± 7.3	26.1 ± 5.1	1.199	0.238
Education (years)	13.8 ± 1.8	14.4 ± 1.8	−1.107	0.275
RPM score	49.0 ± 5.9	54.1 ± 5.4	−2.725	0.01
PANSS scores				
Total	56.5 ± 16.8			
Positive	13.4 ± 3.9			
Negative	16.2 ± 3.7			
General	29.9 ± 6.1			
CPZ equivalent dose (mg)	369.4 ± 354.9			
Correct response rate (%)				
Overall	94.2 ± 5.0	96.7 ± 2.9	−1.806	0.083
Congruent positive	95.2 ± 4.8	97.5 ± 2.8	−1.757	0.088
Congruent negative	92.6 ± 7.4	96.1 ± 3.4	−1.925	0.062
Incongruent positive	94.8 ± 5.6	96.8 ± 5.2	−1.090	0.283
Incongruent negative	94.1 ± 6.2	96.3 ± 3.0	−1.318	0.201
Reaction time (ms)				
Overall	714.9 ± 51.9	676.2 ± 39.8	2.507	0.018
Congruent positive	705.0 ± 50.9	661.8 ± 51.0	2.574	0.014
Congruent negative	735.5 ± 52.5	687.4 ± 48.1	2.886	0.007
Incongruent positive	716.0 ± 64.4	675.1 ± 40.9	2.263	0.032
Incongruent negative	703.2 ± 64.6	680.7 ± 51.2	1.157	0.256

*Note.* All data except sex are given in mean ± standard deviation.

RPM: Raven's Progressive Matrices; PANSS: Positive and Negative Syndrome Scale; CPZ: chlorpromazine.

### 2.2 Behavioral task

**Figure 1:**
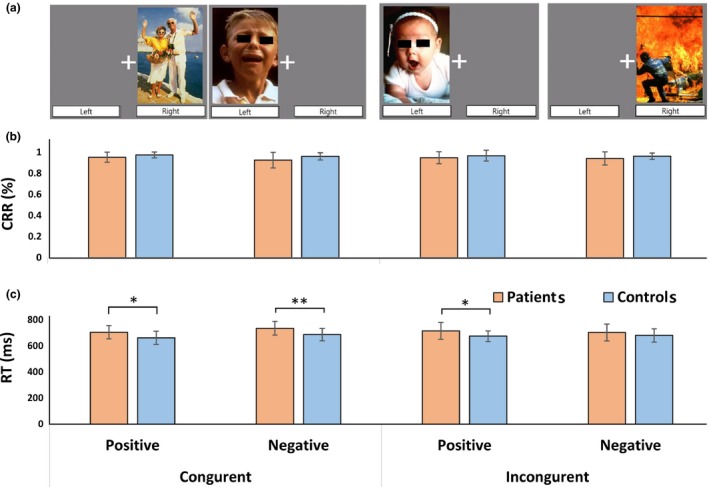
Behavioral task and performance. (a) In the emotional Simon task, participants are asked to press the right or left button in response to a positive or negative emotion, respectively. (b) Correct response rate (CPR) showed no difference between patients with schizophrenia and controls in all conditions. (c) In most conditions, patients showed significantly longer reaction time (RT) than controls. Error bars represent standard deviations. *p <* *0.05, **p <* *0.01

Participants performed a modified Simon task, in which positive or negative emotional pictures were presented serially on the left or right side of the screen (Figure 1a). A total of 80 pictures consisting of 40 positive and 40 negative emotional depictions were selected from the International Affective Picture System (IAPS; Lang, Bradley, & Cuthbert, 1999), with mean IAPS valence scores of 7.48 ± 0.40 and 2.79 ± 0.66, respectively. Emotional pictures were displayed on the ipsilateral or contralateral side of a matched index finger. The participant's task was to press a button with the left or right index finger in response to a positive or negative emotion regardless of the location of the picture. Trials consisted of four types of stimuli: a positive or negative emotional value and a congruent or incongruent position consistency between stimulus and pressing finger when an emotional picture was presented on the ipsilateral or contralateral sides of a matched index finger. The task sequence was composed of a rapid event‐related design in which each trial had a fixed duration of 1.5 s regardless of the participant's response; inter‐trial intervals varied from 0.5 to 4.5 s. The task was divided into two sessions, each with 160 trials: 40 positive congruence, 40 positive incongruence, 40 negative congruence, and 40 negative incongruence trials (for details, Chun et al., 2017).

### 2.3 Functional imaging

MR scanning was performed using a 3.0T Philips INTERA scanner (Philips Medical Systems, Best, Netherlands). Functional images were acquired using a T2*‐weighted gradient echo‐planar imaging sequence (31 slices of 3.5‐mm thickness with no gaps; repetition time [TR] = 2 s, echo time [TE] = 30 ms, flip angle = 90°, image matrix = 124 × 124, field of view = 220 mm, voxel size = 2 × 2×2 mm^3^) with an in‐plane resolution of 1.719 × 1.719 mm. Imaging slices were obtained at a tilted angle of 30° from the anterior commissure–posterior commissure line to minimize signal loss in the orbitofrontal cortex. Structural images were acquired using a 3D T1‐weighted gradient echo sequence (170 slices, TR = 9.692 ms, TE = 4.59 ms, image matrix = 224 × 224).

### 2.4 Statistical analyses

Demographic data were compared between the groups using a Student's *t* test. Task performances (correct response rate and reaction time) were measured automatically and analyzed separately using analysis of covariance (ANCOVA) with group (patients/controls), emotion (positive/negative), and conflict (congruent/incongruent) as factors and RPM score as covariate. Post hoc analysis using a Student's *t* test was performed for comparison between groups in each emotion and conflict condition.

Image processing and statistical tests were performed using Statistical Parametric Mapping 12 (SPM12). After discarding the first six images from the dummy scan in each session, the remaining 220 images were used for further preprocessing. Differences in slice acquisition time of the interleaved sequence were corrected, and realignment was performed to correct artifacts created by head motion. Corrected images were coregistered on the T1‐weighted image of the same participant. T1‐weighted images were normalized to the standard T1 template, and the resulting transformation matrices were applied to the coregistered functional images. Functional data were smoothed with a Gaussian kernel of 6‐mm full‐width at half‐maximum (FWHM).

Preprocessed data were analyzed using a general linear model. Experimental trials were modeled separately using a canonical hemodynamic response function for individual data. Multiple linear regression using a least‐squares approach was used to obtain parameter estimates (Friston, Frith, Frackowiak, & Turner, 1995). These estimates were then analyzed by testing specific contrasts using the participant as a random factor. Images of parameter estimates for the four different conditions were created in the primary analysis, during which individual realignment parameters were entered as a regressor to control for head movement. All images including those from missing or inaccurate trials were included because they were considered to reflect conflict control.

For the secondary analysis, first‐level contrasts were entered into ANCOVA using a flexible factorial model with group (patient/control), emotion (positive/negative), and conflict (congruent/incongruent) as factors and RPM score as covariate. All reported regional clusters survived at a threshold of a corrected *p* < 0.05, which corresponds to the family‐wise error corrected significance at the cluster level with a cluster‐defining threshold of *p *<* *0.001. Estimated smoothness (or spatial correlation of the results) was estimated as [FWHM in mm] = 9.1, 8.8, and 8.6. For post hoc analysis, regions of interest (ROIs) were defined as whole clusters showing the significant main effect or interaction. The contrast estimate in ROIs was calculated using MarsBaR (version 0.42, http://marsbar.sourceforge.net/). Pearson correlations were calculated to analyze the relationship of regional beta value with task performance and clinical score in each group. Chlorpromazine‐equivalent doses of antipsychotics were used as a covariate to control for the effect of medication in the patient group.

To provide additional information, we conducted psychophysiological interaction (PPI) analysis (Friston et al., 1997). The seed was defined as a 6‐mm sphere centered at a voxel with the peak statistic within the cluster that showed the significant main effect or interaction in the flexible factorial model. In this analysis, we made contrasts using the positive congruent condition as a control. Given that more intense responses in a situation with strong evaluative input are induced by a negative than by a positive system (Cacioppo, Gardner, & Berston, 1999) and by incongruent than by congruent stimuli (Kitada, Sasaki, Okamoto, Kochiyama, & Sadato, 2014), the positive congruent condition was considered to have the least emotional and cognitive load among the four conditions. As a result, three loading contrasts such as cognitive‐only (positive incongruent—positive congruent), emotional‐only (negative congruent – positive congruent), and cognitive and emotional (negative incongruent – positive congruent) were considered for further evaluation. Voxel‐wise connectivity in each contrast was counted to determine a group difference, and significant clusters were defined at a threshold of family‐wise error corrected *p *<* *0.05. A post hoc test to examine the detailed group difference was based on the connectivity strength, which was extracted in the significant clusters using MarsBaR. Pearson correlations with controlling for chlorpromazine‐equivalent doses of antipsychotics were calculated to analyze the relationship between the connectivity strength and clinical score.

## 3 RESULTS

### 3.1 Behavioral response

As shown in Table 1, compared with controls, patients showed no significant difference in overall rate of correct responses, but significantly longer overall reaction time (*t *=* *2.507, *p *=* *0.018). Correct response rate and reaction time showed no significant interactions for cognitive conflict × group, emotion × group, and cognitive conflict × emotion × group.

### 3.2 Brain activation

**Figure 2:**
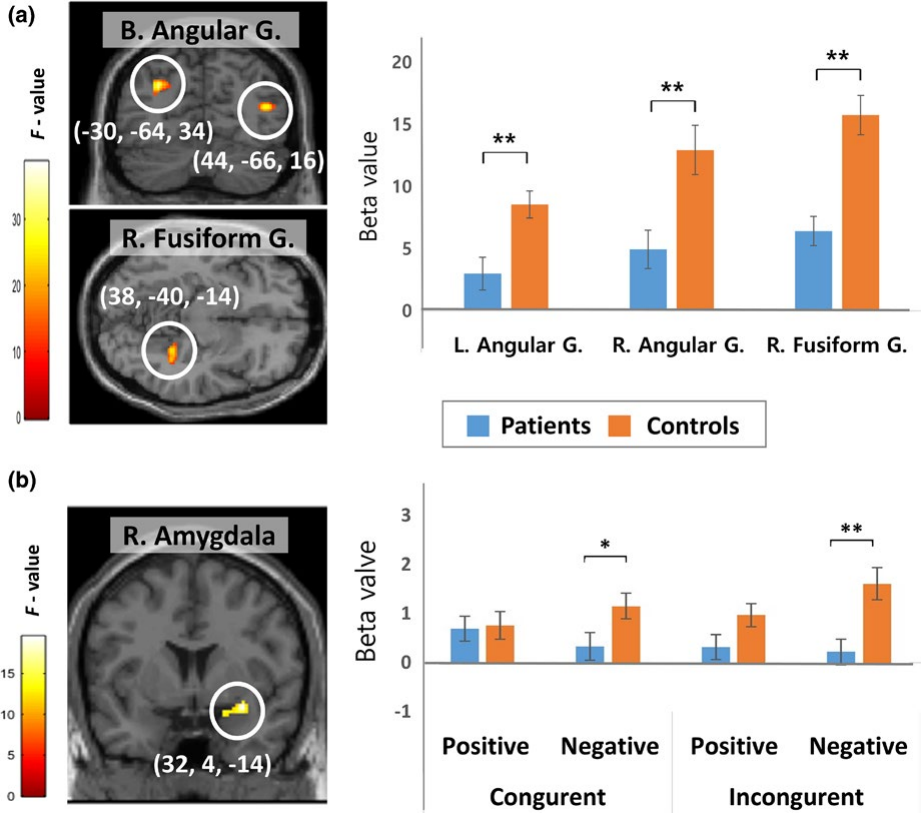
Brain regions showing the main effect of group (a) and significant group × emotion × conflict interaction (b) during the emotional Simon task. Activities in the bilateral (B.) angular gyrus (G.) and right (R.) fusiform gyrus were significantly lower in patients than in controls. The right amygdala was the only region showing a significant interaction; the contrast estimate was extracted from the peak cluster and plotted for each condition in each group. Error bars represent standard errors. *p <* *0.05, **p <* *0.01

As shown in Table 2, brain regions showing the main effect of group were the bilateral angular gyri and right fusiform gyrus. A post hoc test showed that compared with controls, patients exhibited significantly decreased activity in all of these regions (Figure 2a). There was no brain region showing significant group × conflict or group × emotion interaction, but the significant group × emotion × conflict interaction was observed in the right amygdala. Compared with controls, patients exhibited significantly lower right amygdala activity in the negative congruent (*t *=* *−2.168, *p *=* *0.036) and negative incongruent condition (*t *=* *−3.273, *p *=* *0.002), but not in the positive congruent or positive incongruent condition (Figure 2b). Control participants showed higher activity in the negative incongruent condition than in the negative congruent condition, whereas patients exhibited the opposite pattern. These group differences were not statistically significant, but appeared to be the source of significant three‐way interaction. Regional activities in the clusters showing significant results showed no correlation with behavioral performances including correct response rate and reaction time and PANSS subtotal scores.

**Table 2:** Brain regions showing the main effect of group and group interaction

	F	MNI Coordinates			Nvox
		*x*	*y*	*z*	
Main effect of group					
Angular gyrus, left	38.6	−30	−64	34	320
Angular gyrus, right	26.5	44	−66	16	87
Fusiform gyrus, right	24.2	38	−40	−14	148
Group*emotion interaction					
None					
Group*conflict interaction					
None					
Group*emotion*conflict interaction					
Amygdala, right	19.5	32	4	−14	152

*Note.* All reported regional clusters survived at a threshold of a corrected *p* < 0.05, which corresponded to the family‐wise error corrected significance at the cluster level.

MNI: Montreal Neurological Institute; Nvox: number of voxels.

### 3.3 Functional connectivity

**Figure 3:**
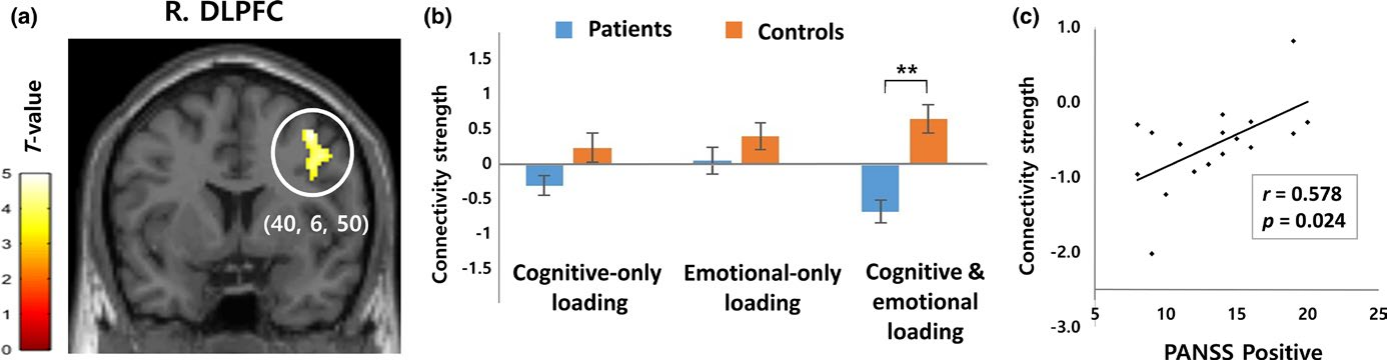
A brain region showing a significant difference in functional connectivity with the right amygdala between patients with schizophrenia and controls. A) The connectivity strength between the right amygdala and right dorsolateral prefrontal cortex (R. DLPFC) was significantly reduced only in the cognitive and emotional loading contrast (negative incongruent—positive congruent). B) The connectivity strengths between the two regions were plotted to show the group difference in each contrast. C) The connectivity strength in the cognitive and emotional loading contrast and the Positive and Negative Syndrome Scale (PANSS)—Positive scores showed a significant positive correlation in patients with schizophrenia. Error bars represent standard errors. **p <* *0.01

All brain regions showing significant group difference in activity and interaction in the flexible factorial model were used as a seed region for PPI analysis to analyze group differences in functional connectivity. When the seeds were the bilateral angular gyri and right fusiform gyrus, there was no significant group difference in any contrasts. When the seed was the right amygdala, there was no significant group difference in the cognitive‐only and emotional‐only loading contrasts, but the cognitive and emotional loading contrast produced a significant group difference in the right DLPFC (cluster‐level pFWE‐corr = 0.007; *x* = 40, *y* = 6, *z* = 50) (Figure 3a). A post hoc test showed significantly lower connectivity in the cognitive and emotional loading contrast in patients than in controls (*t *=* *−5.154, *p *<* *0.01), but no group difference in the cognitive‐only or emotional‐only loading contrast (Figure 3b). The connectivity strength between the right amygdala and right DLPFC in the cognitive and emotional loading contrast was significantly correlated with PANSS positive (*r *=* *0.578, *p *=* *0.024; Figure 3c) and general (*r *=* *0.627, *p *=* *0.012) scores, but not with PANSS negative score.

## 4 DISCUSSION

In our investigation of the abnormal relationship between emotion and conflict control in patients with schizophrenia with fMRI, the right amygdala was the only region that showed significant emotion × conflict × group interaction. This result suggests that amygdala dysfunction may be a key component of this abnormal interaction in schizophrenia. Patients showed less right amygdala activity than in healthy controls under negative emotion, but not under positive emotion. In particular, the group difference was amplified under the negative incongruent condition in which cognitive conflict is resolved under negative emotion, suggesting that healthy controls properly involve the amygdala in the resolution of interference induced by negative emotion, but patients with schizophrenia do not.

Our finding is consistent with a previous report of reduced amygdala modulation in response to varying emotional stimuli in patients with schizophrenia (Pinkham et al., 2011). Patients with schizophrenia also showed hypoactivity in the amygdala when performing emotion labeling in the task of imagining sadness feelings or showing unpleasant stimuli (Johnston, Stojanov, Devir, & Schall, 2005; Mier et al., 2014; Pinkham, Gur, & Gur, 2007). Therefore, reduced amygdala activity may impair effective use of emotional and cognitive resources to resolve conflict. On the other hand, it should be noted that other previous reports have shown amygdala hyperactivity during emotional processing or resting state in patients with schizophrenia (Escartí et al., 2010; Pinkham et al., 2015). This discrepancy may be attributed to a difference in cognitive loading involved in emotional tasks. For example, task difficulty may modulate the impact of emotional stimuli on regional responses associated with conflict control (Jasinska, Yasuda, Rhodes, Wang, & Polk, 2012).

Reduced amygdala activity responding to negative emotional stimuli in schizophrenia can disturb the prefrontal involvement in conflict situations. In our study, functional connectivity between the right amygdala and right DLPFC was reduced in patients, suggesting that reduced amygdala–DLPFC connectivity may underlie abnormal emotion–cognition interaction in schizophrenia. This result is consistent with a recent finding that the amygdala and medial PFC showed negative functional coupling during emotion perception in patients with schizophrenia and positive functional coupling in controls (Bjorkquist et al., 2016). It should be noted that abnormal amygdala–DLPFC connectivity was observed in patients in the cognitive and emotional loading contrast, but not in the only cognitive or emotional loading contrast. This finding suggests that the interaction of negative emotion and cognitive conflict is particularly important in the circuital dysfunction.

It has been suggested that the DLPFC plays a pivotal role in the dynamic tuning of executive control and conflict‐induced behavioral adaptation (Mansouri, Tanaka, & Buckley, 2009). The DLPFC is also involved in the link between emotion and goal‐directed behavior, and its dysfunction in schizophrenia may contribute to defective processes related to emotion–cognition interaction (Becerril & Barch, 2011; Ursu et al., 2011). Interestingly, the right amygdala–right DLPFC connectivity strength in the cognitive and emotional loading contrast was positively correlated with the severity of positive symptoms. This result agrees with a previous finding that top–down endogenous DLPFC–amygdala connectivity during implicit processing of affective stimuli was reduced in patients with schizophrenia, and the connection significantly influenced the severity of psychotic symptoms (Vai et al., 2015). Taken together, a disconnection between these two brain regions may be related to the symptomatic outcome of schizophrenia. However, given that the DLPFC receives minimal direct projections from the amygdala in contrast to the orbitofrontal cortex and medial PFC, the influence of the amygdala to the DLPFC is likely to be indirect and may be mediated by the ACC or posterior orbitofrontal cortex (Ray & Zald, 2012).

Contrary to our hypothesis, the main effect of group was not found in the PFC or ACC. The behavioral finding that patients’ correct response rate was equal to controls may reflect this lack of difference in PFC or ACC activity. State anxiety impacts the interaction between emotion and cognition via the anterior insula (Choi, Padmala, & Pessoa, 2012), but an abnormal response in this region was not observed in our patients. Instead, patients showed reduced activity in the bilateral angular gyri and right fusiform gyrus while performing the emotional Simon task. Reduced angular gyrus activity in schizophrenia has been observed during facial emotion discrimination (Reske et al., 2009; Streit et al., 2001) or social cognition (Thakkar, Peterman, & Park, 2014). Patients with schizophrenia have also shown neural deficits in the fusiform gyrus during emotional face processing (Gur et al., 2002; Li, Chan, McAlonan, & Gong, 2010; Wolf et al., 2015). Consistent with these studies, we observed reduced angular and fusiform activity in patients induced by pictures which included human faces in various social situations.

Although reaction time was longer in patients than in controls, there was no group difference in correct response rate. This result may be attributed to sufficient time for reaction and low task difficulty in our study. Because emotion was assessed only as positive or negative using emotionally well‐defined pictures, the task might have been easy for both patients and controls. Our result differs from previous studies which reported that patients with schizophrenia performed worse than controls across most emotional or social tasks (Kohler, Walker, Martin, Healey, & Moberg, 2010; Savla, Vella, Armstrong, Penn, & Twamley, 2013). However, some investigators have also suggested that patients with schizophrenia have no increased conflict effects when controlling for various potential confounders and thus have intact conflict control (Smid, Bruggeman, & Martens, 2016). Although patients’ task performance was similar to controls in our study, this does not mean that brain processing was equally effective in the two groups. Even in the absence of behavioral or activation differences, patients with schizophrenia also demonstrated weaker amygdala–DLPFC coupling, particularly during negative distraction (Anticevic et al., 2012). Task performance can be intact in patients with schizophrenia when impaired top–down control is compensated by upregulation of task‐relevant regions (Cieslik et al., 2015). This compensatory functional change may also explain why there were different amygdala responses among the four conditions despite absence of significant interaction between emotion and conflict control in behavioral results. In this aspect, the group interaction effect may reflect a difference in the effectiveness of brain processing.

This study has several limitations. First, effects of medications were not ruled out. Antipsychotic drug effects in schizophrenia may include global brain functions and connectivity (Abbott, Jaramillo, Wilcox, & Hamilton, 2013; Kraguljac et al., 2015). In particular, antipsychotics could alter neural activity and functional connectivity of the amygdala (Rasetti et al., 2009) and different antipsychotics could have different effects on amygdala activity during emotional processing (Surguladze et al., 2011). Second, educational level was controlled, but intelligence was not. To control this factor, we used RPM score as a covariate in analysis. Third, we did not measure whether there was a difference in arousal between the control and patient groups. Even if patients felt negative emotions as did controls, the intensity of their emotions could be lower and associated with a difference in brain activity. Fourth, the study was conducted on a relatively small number of participants. Last, as our visual stimuli did not include neutral emotion pictures, we were forced to make different contrasts between activation analysis and PPI analysis.

In summary, we addressed the influence of emotional stimulus on conflict control and its neural basis in schizophrenia. Although patients with schizophrenia did not show impaired accuracy in the emotional Simon task, imaging analysis revealed significant interaction among group, emotion, and conflict in the right amygdala. Patients’ regional activity in this region was particularly reduced in the negative incongruent condition, and functional connectivity of this region with the DLPFC was also reduced. These results suggest that negative emotion interferes with cognitive conflict resolution in patients with schizophrenia and that dysfunctional amygdala–DLPFC connectivity play an important role in this deficit. Based on these findings, interventions targeting conflict control under negative emotional influence may promote cognitive rehabilitation in patients with schizophrenia.

## CONFLICT OF INTEREST

All authors declare that they have no conflict of interests.
